# An Intraoral Epitheloid Hemangioendothelioma Masquerading Clinically as Pyogenic Granuloma

**Published:** 2015-03

**Authors:** Bhargavan Sarojini Sreenivasan, Majo Ambooken, Mayeesh Radhakrishna, Joseph Sebastian

**Affiliations:** 1 Department of Oral Pathology and Microbiology, Mar Baselios Dental College, Kothamangalam, Ernakulam, Kerala. India;; 2 Department of Periodontics, Mar Baselios Dental College, Kothamangalam, Ernakulam, Kerala, India

**Keywords:** Epithelioid hemangioendothelioma, Neoplasms, Vascular neoplasm

## Abstract

Epitheloid hemangioendothelioma (EHE) is an uncommon angiocentric neoplasm of intermediate malignant potential. This tumor is frequently seen in the lungs and liver, but its presentation in the oral cavity is rare. In the oral cavity, gingival region is the common sites of occurrence. We report a rare case of epitheloid hemangioendothelioma in a 48-year-old male, presenting as a growth in the upper anterior gingiva of five months duration along with a review of its clinicopathological and immunohistochemical characteristics.

## Introduction

The term hemangioendothelioma was introduced by Borrmann who first proposed the concept of vascular neoplasms with intermediate or low malignant potential. Three histological types of hemangioendothelioma are; Kaposiform, Hobnail (or Dabska-retiform), and Epithelioid.^1^ Epithelioid hemangioendothelioma (EHE) is a very rare vascular neoplasm. It is described as an angiocentric neoplasm characterized by neoplastic proliferation of epithelioid endothelial cells, showing eosinophilic vacuolated cytoplasm, and occasionally, fusiform cells. The cell proliferation is usually arranged as short anastamosing cords, solid lobules or lining primitive appearing vascular channels, with erythrocytes occasionally seen in the lumina. Frequently, the tumor cells are arranged within a fibromyxoid stroma.^2^

The common site of occurrence of EHE is the lung, liver, soft tissue, and bone.^3^ Occurrence of epitheloid hemangioendothelioma in oral cavity is rare.^2^ In oral cavity, it is commonly seen in palate and gingival.^4^ To the best of our knowledge, the review of the English literature revealed a total of 30 intraoral epitheloid hemangioendothelioma reported cases.^1^^,^^4^^,^^5^ Some of these intraoral tumors have shown local recurrences.^1^ Since only few cases have been reported, the exact clinical behavior of the intraoral EHE is still uncertain.^4^ In this article, we report a case of intraoral epithelioid hemangioendothelioma masquerading clinically as pyogenic granuloma.

## Case Report

**Figure 1:**
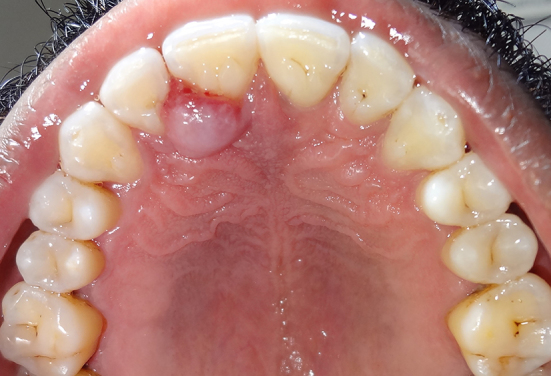
Palatal aspect of the swelling between 21 and 22.

**Figure 2:**
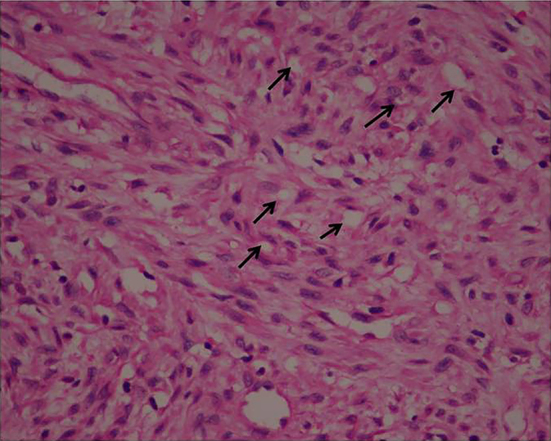
Tumor showing spindle and epitheloid cells with intracytoplamic vacuoles indicated by black arrows (×40).

**Figure 3:**
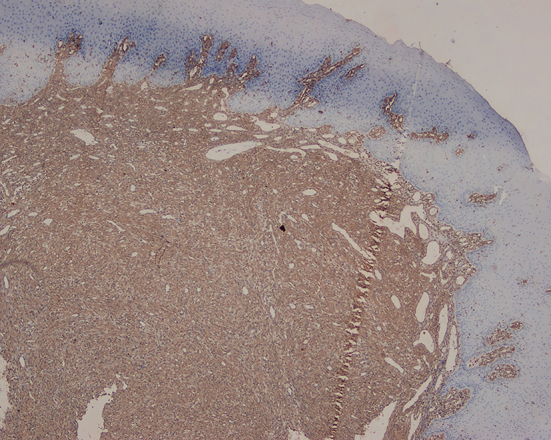
Tumor tissue showing vimentin immuno positivity (×4).

**Figure 4:**
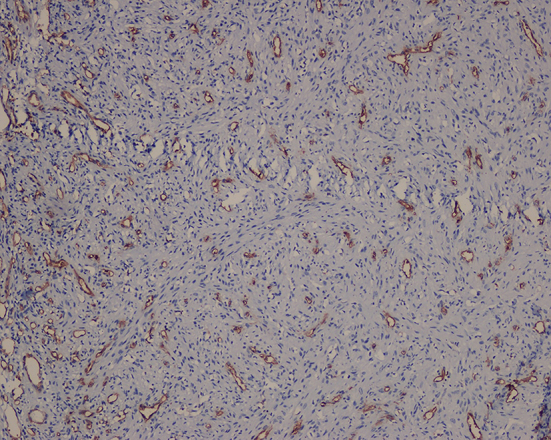
Tumor cells especially the ones forming small vessels and intracytoplamic vacuoles showed strong staining with CD34 (×10).

**Figure 5:**
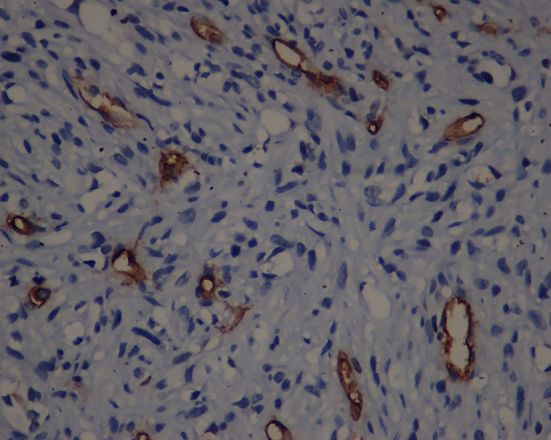
Tumor cells especially the ones forming small vessels and intracytoplamic vacuoles showed strong staining with CD34 (×40).

A 48-year-old man presented with a chief complaint of localized gum enlargement since 5 months. The patient had no relevant medical history. On examination, a small sessile grayish pink growth of size 7×7 mm on the buccal and palatal aspect of interdental gingiva between 21 and 22 regions was found. The swelling was firm and nontender on palpation. The underlying bone showed no evidence of erosion on radiographic examination. Similar swellings were not present in other parts of the oral cavity or skin and lymph nodes were not palpable. The lesion was provisionally diagnosed as pyogenic granuloma (figure 1). The patient underwent routine blood examination prior to biopsy procedure and the results were within normal limits (Bleeding Time: 2 min, Clotting Time: 10 min, Random Blood Sugar: 70 gm/dl, Hemoglobin Count: 16.5 gm/dl, Total Leucocyte Count: 5700 cells/mm3. Differential Count; Neutrophils: 64%, Lymphocytes: 36%, Erythrocyte Sedimentation Rate: 4 mm/hour). An excision biopsy was performed and on microscopic examination, a fibrovascular connective tissue with a circumscribed tumor mass of epitheloid and spindle shaped endothelial cells. The tumor cells comprised predominantly of epitheloid cells. These cells exhibited intracellular vacuoles, which in some foci contained RBCs (figure 2). The immunohistochemical study on the tumor showed that the cells were positive for vimentin (figure 3), and negative for smooth muscle actin (SMA). The neoplastic cells showed strong staining with CD34 especially by the cells forming intracytoplasmic vacuoles (figure 4 and 5).

Based on histopathological and immunohistochemical findings, the lesion was diagnosed as epithelioid hemangioendothelioma. Computerized tomography of the chest and ultrasound of the abdomen was done to rule out any hidden primaries and no abnormalities were detected. The patient is under a regular follow-up for 10 months and there is no evidence of recurrence. We had obtained consent from the patient for publishing this report. 

## Discussion

The epithelioid hemangioendothelioma (EHE) is a rare angiocentric vascular tumor of intermediate malignancy that usually arises in the superficial or deep soft tissues of the extremities. In the head and neck area, EHE is commonly seen in the submandibular region.^5^ Ellis and Kratochvil in 1986 first reported EHE in the oral cavity.^6^ Most common oral site was in the gingiva.^7^ It can occur at any age and they are predominantly seen in fourth and fifth decades of life. There is no sex predilection or associated predisposing factors for EHE.^8^

Most cases were clinically diagnosed as benign entities like pyogenic granuloma, fibroma, peripheral giant cell granuloma, peripheral ossifying fibroma, inflammatory fibrous hyperplasia and necrotizing ulcerative gingivitis.^7^ The case reported here was also diagnosed clinically as pyogenic granuloma.

The microscopic differential diagnosis includes carcinoma, angiosarcoma and Hemangio- pericytoma.^9^ Vimentin will be negative for carcinomas.^1^ In our case the tumor cells were vimentin positive and thus the possibility of being a carcinoma as the diagnosis was ruled out. Epitheloid angiosarcomas present with marked cytologic atypia and pleomorphism and high mitotic rate.^8^ In our case, the tumor was well circumscribed, lacked mitotic figures and cytological atypia, excluding the possibility of angiosarcoma. Tumor cells in hemangiopericytoma are positive for SMA and in our case, the tumor cells were SMA negative ruling out hemangiopericytoma from the histopathological differential diagnosis.^9^ The tumor cells were positive for vascular marker CD34 confirming that they are endothelial cells.^3^ Majority of EHEs are clinically diagnosed as benign lesions. It’s important to make a proper histopathological examination and come to a correct diagnosis, since it is a neoplasm with malignant biological behavior, intermediate between the hemangioma and conventional angiosarcoma.^3^^,^^10^

It is reported that when EHE’s present with increased mitotic figures, cellular atypia, spindle tumor cells, metaplastic bone formation and areas of necrosis; they can behave in a more aggressive manner.^1^ The case reported here showed no relevant cellular atypia or mitotic activity and was predominantly composed of epitheloid cells suggestive of a less aggressive variant of the tumor.

Epitheloid hemangioendothelioma exhibits different biological behavior depending upon the anatomical position. There are no consistent clinical or histological criteria for predicting the behavior and prognosis of intraoral EHE.^4^ According to Chi et al., the average age of occurrence of intraoral EHE is 28 years, but in our case the patient was in his 5^th^ decade.^9^ When the cases of intraoral EHE reported up to 2012 were analyzed, the common site of occurrence was gingiva (13/30 cases) ( table 1).^1^^,^^2^^,^^4^^,^^5^ In our case, also the site of occurrence of the tumor was the same. Manuel-Antonio Gordón-Núñez et al. analyzed 27 reported cases of intraoral EHE of which 23 cases (85.2%) had clinical follow-up information. Only eight lesions (29.6%) recurred locally and there were no reported local or distant metastasis. These findings suggest that intraoral EHE’s are less aggressive in nature.^1^

**Table 1:** Clinical data for cases of intraoral oral EHE reported in English literature

**Serial No **	**Author**	**Age**	**Sex**	**Localization**	**Clinical and radiographic history**	**Follow-up**
1	Wesley et al.^11^	18	F	Mandibular gingiva	Reddish erosive lesion, (34 to 36), bone resorption	2 years SFL
2	Ellis et al.^6^	13	F	Maxillary gingiva	Swelling, pink, tooth mobility, 4 years	6 years SFL
3	Ellis et al.^6^	4	F	Mandibular gingiva	Tooth mobility, bone resorption	NI
4	Moran et al.^12^	25	F	Palate	Swelling, 1.0 cm, 1 year	21 months SFL
5	de Araujo et al.^13^	4	M	Mandibular gingiva	Swelling, ulceration, tooth mobility, 9 months	NI
6	Marrogi et al.^10^	45	M	Maxillary gingiva	Erythematous lesion, 1.5 cm	3,6 months Rec
7	Marrogi et al.^10^	36	F	Tongue	Painful nodules, 0.2 cm, 2 months	17 months SFL
8	Flaitz et al.^14^	7	F	Mandibular gingiva	Reddish swelling, 1.5 cm, tooth mobility, bone destruction	52 months SFL
9	Hamakawa et al.^15^	76	F	Mandibular anterior region	Submucous swelling, soft, 4.5 cm, bone destruction	6 years SFL
10	Orsini et al.^7^	18	F	Buccal mucosa	Asymptomatic swelling, 1.5 cm, 7 months	9 months Rec
11	Ramer et al.^16^	32	M	Maxilla	Swelling, 3.5 cm	6 months Rec
12	Molina Palma et al.^17^	65	F	Tongue	Swelling, 0.5 cm, 2 months	21 months SFL
13	Machalka et al.^18^	65	M	Jaw	Swelling at the anterior region of jaw, tooth mobility	4.8 years Rec
14	Anderson et al.^19^	18	F	Lower lip	Asymptomatic swelling, 6 months	4 months Rec
15	Chi et al^9^	28	F	Maxillary gingiva	Purple swelling, 0.6 cm	8 months SFL
16	Chi et al.^9^	23	F	Jaw	2.0 cm, bone destruction	NI
17	Sun et al.^20^	12	M	Maxillary gingiva	Ulcerated swelling, 3.0 cm, 3 months, bone destruction, tooth mobility	6 months SFL
18	Sun et al.^20^	53	M	Buccal mucosa	Swelling, 1.5, 6 months	9 months Rec
19	Sun et al.^20^	17	M	Tongue	Soft swelling, 0.5 cm, 2 months	18 months SFL
20	Sun et al.^20^	52	F	Upper lip	Purple swelling, 2.0 cm, 1 year	3 years SFL
21	Sun et al.^20^	21	M	Tongue	Reddish swelling, 0.5 cm, 2 months	2 years SFL
22	Sun et al.^20^	34	M	Tongue	Swelling, 1.0 cm, 4 months	6 years SFL
23	Sun et al.^20^	11	M	Mandibular gingiva	Painful swelling, 2.0 cm, 1 month, bone destruction, tooth mobility	8 years SFL
24	Sun et al.^20^	46	M	Tongue	Reddish swelling, 1.2 cm	4 months Rec
25	Sun et al.^20^	6	M	Floor of mouth and tongue	Reddish swelling, 7.0 cm, 6 months	2 years SL
26	Mohtasham et al.^5^	9	M	Maxillary gingiva	Ulcerated reddish swelling, asymptomatic, 1.0 cm, 6 months	1 year, Rec
27	Gordón-Núñez et al.^1^	17	F	Mandibular gingiva	Swelling, pink, 2,0 cm, 1 year	9 months, SFL
28	Nooshin Mohtasham et al.^5^	9	M	Maxillary gingiva	Pedunculated, reddish swelling; 1 cm; 6 months.	NI
29	Bhari Manjunatha et al.^4^	20	M	Floor of the mouth	Pedunculated, reddish swelling; 3**×**4 cm; 6 months	NI
30	Manuel-Antonio et al.^1^	17	F	Mandibular gingiva	Pedunculated, exophytic pink swelling; 1year	14 months SFL

M: Male; F: Female; NI: No information; SFL: Survival free of lesion; SL: Survival with lesion; Rec: Recurrence

The treatment of choice for epitheloid hemangioendothelioma is complete surgical excision along with wide margins. The follow up data of Intraoral EHE reported until 2012 shows that 13% cases had local recurrences before 10 months (table1) but our patient had a disease free period of 10 months. Machalka et al. in 2003 reported intraoral EHE with recurrence after a disease free period of 8 years, so a long term follow up is recommended.^1^^,^^4^^,^^5^^,^^9^

## Conclusion

Intraoral EHE is an intermediate malignancy, which has a tendency for local recurrence and distant metastasis. It is often diagnosed clinically as other benign lesions. Hence, clinicians should be aware of pathological features and behavior of this lesion in order to ensure prompt diagnosis and appropriate management for the patient. Since very few reported cases of intraoral epithelioid hemangioendothelioma are available, the exact biological behavior of the lesion is yet to be ascertained. 
